# The intersectional structural violence that Latinx health students are experiencing and its consequences

**DOI:** 10.3389/fpubh.2026.1861040

**Published:** 2026-06-11

**Authors:** Yesnely Anacari Flores, Don Operario

**Affiliations:** Rollins School of Public Health, Emory University, Atlanta, GA, United States

**Keywords:** DEI (diversity-equity-inclusion), health equity, immigration, public health, racism

## Abstract

Latinx public health students occupy a uniquely vulnerable position at the intersection of racialized, immigrant, and academic identities-one that is increasingly threatened by a rapidly intensifying anti-immigrant, anti-diversity-equity-inclusion, and anti-public health climate. Using an intersectional structural violence framework, this perspective examines how contemporary anti-immigrant, anti-DEI, and anti-public health policies affect Latinx students pursuing public health education in the United States. By synthesizing emerging policy developments and related public health scholarship, we illustrate how multiple systems of exclusion may converge to shape educational, psychological, and professional outcomes of this population. We outline institutional and professional strategies to mitigate harm and support the development of a diverse, equitable, and sustainable public health workforce.

## Introduction

People of Hispanic or Latin American descent (hereafter, Latinx individuals) are the largest racial and ethnic group in the United States, numbering approximately 68 million individuals as of 2024 (1). Within the health professions, 18% of individuals identify as Latinx, with applications from Latinx students to Master of Public Health (MPH) programs surged by 60% during the onset of the pandemic (2, 3). Over the past decade, there has been progress toward promoting inclusion, diversity, and safety for students of color in public health. These efforts included increased grant opportunities for diversity-focused research, scholarships to support students of color, and institutional declarations recognizing racism as a public health issue (4–6). However, since January 2025, the Latinx community, along with the broader field of public health, has faced a rapidly intensifying pattern of political and policy challenges including efforts to dismantle these initiatives. The simultaneously targeting of the immigrant community and the field of public health highlights the intersectional vulnerability that Latinx public health students face.

This perspective applies an intersectional structural violence framework to examine how contemporary anti-immigrant, anti-diversity-equity-inclusion (DEI), and anti-public health policies affect Latinx students pursuing public health education in the United States. We argue that these students occupy a vulnerable intersection of identities within a rapidly changing sociopolitical environment due to their positionalities as students, members of racialized communities, and (for many) membership within immigrant families. By synthesizing emerging policy developments and related public health scholarship, this paper illustrates how multiple systems of exclusion may converge to shape educational, psychological, and professional outcomes among Latinx public health students. We conclude by outlining institutional and professional strategies to mitigate harm and support the development of a diverse and equitable public health workforce.

## Applying an intersectional structural violence framework to Latinx public health students

While the Latinx community is not interchangeable with the immigrant community, there is substantial overlap between these populations. Approximately 5.8 million students enrolled in higher education are of immigrant origin, meaning they have a parent with immigrant status (7). Latinx students, regardless of their own immigration status, may experience compounded effects of structural violence due to their status as students, racial/ethnic minorities, and members of immigrant communities. This current sociopolitical moment highlights the intersectional identities of many Latinx students and the myriad ways in which systems of oppression (i.e., racism, ethnocentrism, and classism) can impact their mental, physical and social wellbeing (8). Intersectional structural violence is a framework that pulls from Crenshaw’s (9) intersectionality theory and Galtung’s (10) theory of structural violence theory to examine how systems and policies intersect and reinforce one another to produce compounded forms of harm. This framework aligns with a growing body of scholarship that applies intersectionality and structural violence as complementary and synergistic approaches for understanding how structural inequities may differentially shape experiences according to individuals’ intersecting social identities and their relative proximity to systems of power, oppression, and violence (11–14). In the context of Latinx public health students, this framework helps illustrate how immigration-related policies, racialized discrimination, educational exclusion, and threats to public health institutions operate simultaneously to shape students’ wellbeing and their educational and professional outcomes.

Since the new administration took office, it has enacted a wave of policies widely viewed as anti-immigrant and harmful to Latinx communities, such as the repeal of “sensitive locations,” threats to in-state tuition access, and the dismantling of diversity, equity, and inclusion (DEI) programs, and reduced support for public health research and workforce development (15–17). While some policies have been challenged or blocked through legal challenges, many are currently being implemented or remain under active consideration. These policy changes are likely to have important social, educational, and health implications for Latinx students pursuing careers in public health (18).

Through the lens of intersectional structural violence, these policy shifts can be understood not as isolated events but as interconnected actions operating across multiple domains in students’ lives. The following sections describe how these intersecting conditions shape educational access and safety, increase psychological distress, and threaten the long-term impact of the public health workforce. Given these intersecting challenges, public health institutions must adopt strategic responses to safeguard Latinx students’ wellbeing and ensure their continued educational and professional development. Table 1 summarizes selected intersecting policies and structural conditions (i.e., “attacks”) affecting Latinx public health students.

**Table 1:** Examples of policies and conditions affecting students across intersecting identities in 2025–2026.

Type of attack	Policy or structural condition	Potential mechanisms of harm	Intersecting identities affected
Educational attacks	The repeal of the longstanding “sensitive locations” policy in 2025 allows immigration enforcement near K-12 schools (16).	May heighten fear and anxiety among students and their families, potentially disrupting educational accomplishment (16).	Immigrant community member; racial/ethnic minority
	Citizenship verification requirements and restricted educational access for undocumented individuals (19).	May decrease enrollment among undocumented individuals and increase fear and uncertainty (16).	Immigrant community member; racial/ethnic minority; public health student
	Threatened or actual removal of in-state tuition for many dreamers (20).	May render higher education financially unattainable for many students (20).	Immigrant community member; racial/ethnic minority; public health student
Safety attacks	Overturning of federal protections that limit ICE from gathering information based on race, ethnicity, language or workplace (21).	May increase fears of surveillance, detention, deportation, and discriminatory profiling within immigrant communities (39, 40).	Immigrant community member; racial/ethnic minority
	Intensified ICE raids and immigration enforcement activities across the nation.	May increase fear, anxiety and potential disruptions to students personal and family lives (38, 40).	Immigrant community member; racial/ethnic minority
	Increased surveillance and punitive actions toward student advocates, many of whom are immigrants (32).	May suppress academic freedom and rights to protest, increasing fears of retaliation among student activists (32).	Immigrant community member; racial/ethnic minority
Attacks on Latinx and/or immigrant research	Termination or restriction of Latinx- or immigrant-focused research grants (36).	May discourage Latinx and/or immigrant students from pursuing community-focused scholarship and limit research advancement addressing Latinx and immigrant health.	Immigrant community member; racial/ethnic minority
Attacks on DEI & the public health workforce	Withdrawal of graduate fellowships and research awards associated with DEI language and health equity (36).	May increase financial precarity and narrow professional pathways for Latinx students.	Immigrant community member; racial/ethnic minority
	Saturated workforce application pools due to contraction of governmental public health jobs (i.e., CDC layoffs) (36).	May increase precarious employment for current and recent public health graduates (36).	Public health student
	Exclusion of public health programs from the definition of “professional degrees” under the One Big Beautiful Bill Act (OBBBA) (41).	May reduce access to federal student loan support and further limit affordability of graduate education for Latinx students (41).	Public health student

Policies and structural conditions reflect developments occurring in 2025–2026. Some policies have been challenged, blocked, or overturned through ongoing legal and political processes.

### Educational attacks

Across educational settings, spanning K-12 to colleges and universities, recent policy shifts and legislative proposals have contributed to increasingly precarious environments for many Latinx students and their families, shaping the broader social conditions that influence educational access and attainment over time. The repeal of the longstanding “sensitive locations” policy in 2025 allows immigration enforcement near K-12 schools, heightening fears that may discourage attendance and engagement (16). Meanwhile, the Department of Education’s reclassification of certain postsecondary programs as “federal public benefits” may require students to provide citizenship verification and potentially restrict access for some undocumented students (19).

These pressures extend beyond K-12 settings. Ongoing federal efforts to dismantle state tuition equity policies, illustrated by the repeal of Texas’s Dream Act, further limits pathways to affordable higher education for many Latinx students (17). Furthermore, Latinx students with precarious immigration status, such as those on visas or receiving Deferred Action for Childhood Arrivals (DACA), may experience heightened vulnerability due to visa instability and potential loss of in-state tuition (20). These developments are likely to transform education from sites of opportunity and advancement into sites of exclusion and threat, with long-term consequences for the diversity of the public health workforce at the precise moment field needs it most.

### Safety attacks

In September 2025, the Supreme Court overturned a federal order that had restricted Immigration and Customs Enforcement (ICE) from inquiring into immigration status based on factors such as race or ethnicity, language, or workplace (21). This decision may increase concerns about discriminatory profiling and heighten fear among Latinx students, their families, and their communities (22). At the same time, ICE raids have intensified across the United States. For example, a large-scale raid at a Toyota Hyundai Metaplant in Georgia resulted in the detention of approximately 475 workers (23). Deportations have reportedly quadrupled since mid-September 2025, compared to the same period in 2024 (24). While ICE presence and related forms of legal violence are not new phenomena, the heightened pace, visibility, and scale of recent enforcement actions may contribute to psychological distress among Latinx students, especially those affected through family and community networks.

A growing body of public health scholarship has documented the health harms associated with legal violence targeting Latinx immigrant communities (15, 25–31). Yet this literature often overlooks a critical dimension: many of the public health students and professionals engaged in this work are themselves members of targeted immigrant communities. Some immigrant student activists are getting targeted and surveilled leading to detainment in some cases with no due process (32). Research conducted during the administration’s first term (2017–2021) and its current term has highlighted the psychological toll of anti-Latinx and anti-immigrant rhetoric and actions, particularly those related to forced family separation and deportations, including increased traumatic stress, chronic anxiety, and reduced feelings of safety among Latinx students (22). Policies that restrict educational access and intensify immigration enforcement do not operate in isolation. Rather, they may reinforce one another in ways that amplify an anti-immigrant climate and undermine mental health, educational experiences, and professional trajectories of Latinx students in public health.

### Attacks on Latinx and/or immigrant public health research

Simultaneously, the public health field is experiencing significant political and institutional pressures (33). Research projects focused on Latinx health and immigrant communities have reportedly been defunded or subjected to heightened oversight and scrutiny, while employment opportunities across the field have become increasingly uncertain due to precarity in federal funding (34, 35). These cuts directly narrow opportunities for the development and advancement of the next generation of Latinx public health professionals.

Students of color often align their research or public health work with the needs of their own communities. As a result, these attacks on Latinx-focused and immigrant-themed research may threaten both the scholarly ambitions and career pathways of Latinx students entering an already challenging professional landscape. Many students may receive limited guidance on how to continue conducting community-engaged or equity-focused work without inadvertently increasing risks to the populations they serve. These conditions may contribute to feelings of discouragement and hopelessness among Latinx students regarding whether, and how, to continue moving this work forward (18).

### Attacks on DEI and the public health workforce

Funding cuts further compound these pressures. DEI-related grants have been halted or terminated at a growing number of institutions, affecting students at diverse public health institutions across the country. Graduate fellowships and research awards have also been revoked for including language related to DEI, health equity, health disparities, and Latinx populations (36). Meanwhile, recent public health graduates are entering an increasingly saturated workforce alongside thousands of other public health professionals, many of whom were released unexpectedly from federal employment, thereby intensifying competition for an already shrinking pool of opportunities.

These financial pressures are deepened by the One Big Beautiful Bill Act (OBBBA) (22, 37), a federal budget reconciliation law enacted in July 2025 that reduced funding across several sectors, including food assistance, health care, education, and student financial assistance and loan programs. Under this legislation, MPHs and DrPH programs were excluded from the definition of “professional degrees,” thereby restricting students’ access to higher federal loan borrowing caps. This policy may prove to be particularly consequential for Latinx students, given evidence that a substantial proportion of Latinx graduate students rely on federal loans to finance their education, making a public health graduate degree even less accessible for Latinx students (22, 37). Taken together, these intersecting pressures of political hostility, surveillance, funding insecurity, and workforce precarity leave Latinx public health students at heightened risk for anxiety and depressive symptoms, threatening not only their individual wellbeing but also the long-term health of a field that depends on their presence (22, 37).

## A call to action

Latinx public health students are navigating an increasingly precarious sociopolitical and educational landscape that affects their personal and professional lives. Public health institutions should adopt strategic and proactive actions to support Latinx students’ mental health, safeguard their public health research and applied work, and advocate for health equity within the broader Latinx community.

### Take a public stance to protect students

Public health institutions should publicly denounce discriminatory and anti-immigrant policies that may threaten the safety and wellbeing of students and their communities. Institutional silence may normalize structural violence and minimize the legitimate fears and emotional reactions that Latinx students experience. Institutional public expressions of solidarity and support for Latinx students and other marginalized populations can serve as protective factors against adverse mental health outcomes (36, 38).

### Create strategic institutional protocols to support student safety and wellbeing

Institutions should develop and implement clear protocols in the event of ICE attempts to enter campuses, similar to protocols increasingly adopted in K-12 settings. Institutions should also provide holistic emotional and academic support to students whose family members have been detained or deported, including assessing the impacts of these events on students’ academic performance. Schools and Programs of Public Health should host “know your rights” forums and related educational programming for students and the wider community, leveraging the expertise of Latinx scholars with expertise in immigrant health equity work.

### Sustain and strengthen community partnerships

Community-based organizations have long advanced immigrant and community health equity outside of government-funded structures. Universities should identify meaningful ways to partner with, legitimize, and compensate these organizations for sharing their expertise with students and academic communities. Through these institutional partnerships, students can receive training from Latinx community experts about conducting community-engaged work while protecting Latinx participants through ethical data management and confidentiality, particularly when working with sensitive immigration-related information.

### Intentionally supporting Latinx and immigrant faculty scholarship

Given the persistent underrepresentation of Latinx faculty within public health, institutions should intentionally support and resource Latinx scholars whose expertise is critical to mentoring students and advancing equity-focused scholarship. Such efforts must move beyond symbolic or performative gestures by including meaningful investment and recognition.

### Provide adaptive and novel career support for students

Universities should provide targeted career development support for Latinx public health students and recent graduates, including guidance to help them translate or adapt their public health skills and expertise to meet evolving workforce opportunities during periods of instability within government systems.

## Conclusion

This perspective should be interpreted in light of several limitations. The manuscript is based on emerging policy developments and secondary literature, rather than primary empirical data collected directly from Latinx public health students. In addition, some policy contexts described in this paper continue to evolve through legal and political processes. Nonetheless, this perspective aims to highlight potential implications for Latinx public students based on emerging evidence. Future empirical investigation is needed to measure the consequences of the current climate on Latinx public health students and the future public health workforce more broadly, as well as to document and assess impacts of institutional interventions aimed to support this community.

Latinx public health students are experiencing compounded stress and fear amid an increasingly violent socio-political climate. Viewed through an intersectional structural violence framework, public health institutions have an important responsibility to protect, support, and foster the well-being and safety of Latinx students through strategic actions. Supporting Latinx public health students, especially those who serve or plan to serve the broader Latinx community or address health equity, is not only an investment in individual students but also in the future public health workforce itself. Safeguarding the well-being of these students is an essential act of solidarity that will advance the profession and the goal of achieving public health equity for all.

## Data Availability

The original contributions presented in the study are included in the article/supplementary material, further inquiries can be directed to the corresponding author.
